# Long-term impact of a chronic disease management program on hospital utilization and cost in an Australian population with heart disease or diabetes

**DOI:** 10.1186/s12913-015-0834-z

**Published:** 2015-04-22

**Authors:** G Brent Hamar, Elizabeth Y Rula, Carter Coberley, James E Pope, Shaun Larkin

**Affiliations:** Healthways Inc, 701 Cool Springs Blvd, Franklin, TN 37067 USA; HCF, Level 6, 403 George Street, Sydney, NSW 2000 Australia

**Keywords:** Disease management, Health outcomes research, Hospital utilization, Financial savings, Risk equalization, Australian health policy

## Abstract

**Background:**

To evaluate the longitudinal value of a chronic disease management program, My Health Guardian (MHG), in reducing hospital utilization and costs over 4 years.

**Methods:**

The MHG program provides individualized support via telephonic nurse outreach and online tools for self-management, behavior change and well-being. In follow up to an initial 18-month analysis of MHG, the current study evaluated program impact over 4 years. A matched-cohort analysis retrospectively compared MHG participants with heart disease or diabetes (treatment, N = 4,948) to non-participants (comparison, N = 28,520) on utilization rates (hospital admission, readmission, total bed days) and hospital claims cost savings. Outcomes were evaluated using regression analyses, controlling for remaining demographic, disease, and pre-program admissions or cost differences between the study groups.

**Results:**

Over the 4 year period, program participation resulted in significant reductions in hospital admissions (−11.4%, P < 0.0001), readmissions (−36.7%, P < 0.0001), and bed days (−17.2%, P < 0.0001). The effect size increased over time for admissions and bed days. The relative odds of any admission and readmission over the 4 years were 27% and 45% lower, respectively, in the treatment group. Cumulative program savings from reduced hospital claims was $3,549 over 4-years; savings values for each program year were significant and increased with time (P = 0.003 to P < 0.0001). Savings calculations did not adjust for pooled costs (and savings) in Australia’s risk equalization system for private insurers.

**Conclusions:**

Results confirm and extend prior program outcomes and support the longitudinal value of the MHG program in reducing hospital utilization and costs for individuals with heart disease or diabetes and demonstrate the increasing program effect with continued participation over time.

## Background

Chronic disease and the afflictions that it brings continue to grow around the globe. Australia is no exception; with an ageing population and increasingly common sedentary lifestyles chronic disease continues to grow and account for the majority of morbidity and burden of health. Cardiovascular (CVD) disease and diabetes are two of the most prevalent chronic diseases affecting Australians today. There were approximately one million Australians living with diagnosed diabetes in 2012 [1] and diabetes is the fastest growing chronic condition in Australia, with more than 100,000 Australians newly diagnosed with this disease each year [2,3]. By 2033, if left unchecked, 3.6 million Australians will be afflicted with type 2 diabetes [4]. Cardiovascular disease is the leading cause of death in Australia, claiming 45,600 lives in 2011 (31% of all deaths) [5].

Projected healthcare expenditures for Australia from 2003 to 2033 estimate a 436% increase in healthcare costs related to diabetes, from $1.6 billion (1.9% of total expenditures) to $8.6 billion (3.5% of total expenditures) [4]. Of this enormous increase in direct diabetes-related spending, most ($6.7 billion) is attributed to type 2 diabetes. For this same 2003–2033 time period direct costs from cardiovascular disease will continue to be the most expensive among diseases, with expenditures projected to rise from $9.3 billion (11.0% of expenditures) to $22.6 billion (9.2% of expenditures), less dramatic but still a 142% increase in cost over this 20 year period [4].

Epidemiological research demonstrates that developed societies show a natural trend toward the accumulation of health risk over time for populations and for individuals as they age. With progressive increases in risk prevalence and resulting chronic disease has come greater need and demand for health care. Although an increasing proportion of economic costs are spent on health care, this spending has not manifested in improvement of the overall health of populations receiving this care. These trends indicate the importance of addressing risk early to avoid costs as a result of “sick care” [6].

As a means to help improve the health of Australians, the Private Health Insurance Act 2007 included a key reform allowing health insurers to begin offering programs described as “Broader Health Cover” (BHC) [7]. The intention of the legislation was to give insurers the capability to offer programs that would help keep their covered members healthy and less costly. Chronic disease management programs (CDMPs) were included under BHC, with a focus on improving management of conditions to reduce morbidity and hospital utilization, and delay disease progression; in effect assisting individuals with chronic disease in achieving a healthier and better quality of life [8].

Health care in Australia is funded by way of a ‘mixed system’ of the publicly funded universal healthcare government program (Medicare) that covers all Australians and optional private health insurance. Forty-seven percent of Australians have private health insurance for hospital treatment coverage [9]. By law, private insurers are not allowed to vary insurance premiums based on risk; all individuals pay the same level of insurance premium. A risk equalization process was established that uses ‘risk pools’ to compensate health insurers whose covered population is older, more risky, and costly in comparison to other plans. Recently, concern has been expressed about the sustainability of the current risk equalization system due to the increasing proportion of pooled benefits as the covered population ages. Because the aging population results in overall higher health care spending [10], premiums increase for all covered individuals. The increasing premiums make private coverage less attractive to young, healthy Australians who are needed in the system to offset the older, sicker policy holders [11]. This cycle highlights the need for expanded use of effective programs such as CDMPs to help address rapidly increasing costs.

Chronic diseases and their effects can be prevented, delayed, or better controlled by incorporating healthier lifestyle behaviors such as healthier eating, increasing exercise, reducing stress, and improving individual self-management of conditions. These changes can have a positive impact in reducing hospital admissions and other medical treatments and procedures [12]. Health insurer, The Hospitals Contribution Fund of Australia (HCF) instituted My Health Guardian (MHG) in 2009 as a long-term strategy to improve the health and well-being of covered members. This population health program includes an intensive CDMP with tailored assistance for individuals with chronic disease in managing existing conditions and adopting healthier behaviors. By reducing risk factors associated with disease progression and the onset of comorbid conditions the program aims to avoid acute events stemming from these conditions and improve overall well-being for its members.

The first study on clinical outcomes of MHG, and the only large-scale study evaluating the effectiveness of an Australian CDMP, evaluated the impact of MHG on hospital admissions, readmission and length of stay for members with heart disease and diabetes. Results demonstrated that the MHG program was effective in reducing the occurrence and severity of hospitalization after 12 and 18 months of program participation [13]. The MHG study population, in comparison to disease-matched HCF plan members who were eligible but did not participate in MHG, displayed significant decreases in hospital admissions and readmissions after both 12 and 18 months, and in average length of hospital stay after 18 months. The magnitude of impact increased over time for all three measures.

The current study evaluates the longitudinal value of the MHG program for members with heart disease and/or diabetes. The intent was to confirm prior results and determine if the decreased hospital utilization was sustained over an extended intervention period. Additionally, the study evaluated program generated savings. Such longitudinal evaluation of program effectiveness can provide guidance to health insurers, policy makers and providers when they are considering how to deliver sustainable and scalable programs to improve health and reduce hospitalizations and related costs.

## Methods

### Study design

A quasi-experimental retrospective cohort study was conducted to evaluate MHG impact over 4 years and to determine if the program effects on hospital outcomes, demonstrated in our prior study, are sustained or improved over time. Additionally, hospital costs were assessed to determine the financial impact of the MHG program. The MHG program commenced operation on May 1, 2009. The base period for this study was defined as the year preceding this begin date: May 1, 2008 to April 30, 2009. The remaining time in 2009 was considered a “ramp-up” period during which there was a primary focus on enrolling eligible HCF members in the program. The treatment period of evaluation for this study was from January 1, 2010 to December 31, 2013. Data used in this study included HCF coverage dates, HCF member demographic information, HCF hospital claims records including secondary diagnosis records, and MHG enrollment data. No identifiable patient information was included in the data set.

### My Health Guardian program

The MHG program is a population health and well-being program available free of charge to individuals enrolled in an HCF plan that provides hospital coverage, and who have a qualifying chronic condition. This study focused only on members with documented diabetes or heart disease, as delineated below. The MHG program provides knowledge, support, and tools for behavior change and management of health conditions to improve well-being. All members have access to an online program with health assessments, health actions plans, personalized health support, education, and health behavior tracking. Members with chronic disease, as identified via hospital claims or self-reported health status, are faced with a higher probability of hospital admission and often need more intensive support in managing their conditions. The MHG program provides these members with telephonic support from registered nurses to assist better self-management. These telephonic interactions are guided by a system that provides nurses with patient data, assessments, and individual action plans tailored to patient data and assessment responses. Nurses record information collected on each call in the same system to inform the next member interaction. In addition to nurses’ clinical judgment, there are standard processes and procedures in place as well as standard core condition surveys and individual action plans defined by participant responses and needs. Outbound call frequency to these members is determined by factors including disease severity, current health status, well-being level, and self-management of their condition(s).

### Study participants

Individuals initially eligible for this study were HCF members aged 20 to 89 with continuous coverage in the base period and four-year intervention period. Treatment members had continual enrollment in the MHG program from January 2010 through December 2013. Eligible comparison members showed no documentation of any participation in the MHG program. Demographic information for individuals under 20 or over 89 years of age was not made available to ensure members in these lower frequency age bands could not be identified. Study eligibility also required documented diagnosis of heart disease [coronary artery disease (CAD) or heart failure (HF)] or diabetes in HCF hospital claims and diagnosis files dating back to January 2003. Coding systems available in the hospital claims and used in identification included International Classification of Diseases – 9th and 10th revision diagnosis codes, Commonwealth Medicare Benefits Schedule procedure coding, and diagnosis-related groups hospital payment coding. An additional diagnosis identifier generated by HCF to indicate the reason for claims was also used in some cases to determine diagnoses in cases when standardized codes were not available in the claim. Because this study was a retrospective analysis of a health promotion initiative conducted anonymously, this study was outside the scope of requiring ethics board review or informed consent according to the Declaration of Helsinki (ethical concerns are limited to human subjects research involving identifiable human material and data) and exclusion criteria outlined in the US Code of Federal Regulations (45 CFR §46.101).

### Study group matching

From the study-eligible population, comparable study groups were created using Coarsened Exact Matching (CEM). Using CEM, members from the treatment and comparison groups were exactly matched within a nonparametric framework into distinct strata, or groups matched with respect to a set of shared characteristics and factors (coarsened variables) associated with the outcome of interest. This matching process is optimized to maximally reduce selection bias and variance between study groups, thus minimizing bias in the final estimate of the studied treatment effect. Among matching methods, CEM typically requires exclusion of fewer cases, allowing for a more generalizable study result [14,15].

Matching variables used to create strata in CEM included:

- Gender
- Age group (categorized into 10 year increments: 20–29 through 80–89)
- Base admission count, grouped (0, 1, 2 or more)
- Count of a member’s core diseases (1–9; count of CAD, HF, diabetes, chronic obstructive pulmonary disease (COPD), asthma, chronic kidney disease, end stage renal disease (ESRD), depression, and cancer)
- Disease status (0/1) for each of the study qualifying conditions: CAD, HF, and diabetes

Strata without at least 1 treatment member and 1 comparison member were excluded. After strata assignment CEM weights are generated that account for study group differences with respect to the matching variables. Treatment members were assigned a weight of 1, while comparison members were assigned a weight specific to their stratum and representative of the proportion of members in that stratum. CEM generated weights were used as covariate to adjust results of multivariate statistical modeling of outcomes [16].

The extent of remaining imbalance and heterogeneity between the study groups was assessed using the L1 metric, a nonparametric measure generated in CEM that quantifies inter-group imbalance by comparing relative frequencies of the two groups across each of the strata [17]. Values of L1 close to zero indicate a higher fidelity match with minimal imbalance, whereas an L1 value of 1 indicates complete dissimilarity or disproportionality between the treatment and comparison groups. The L1 measure was used to optimize matching variables to provide a high quality match while retaining a high number of eligible study members.

### Outcomes and statistical methods

Utilization outcomes assessed included hospital admissions, readmissions within 30 days of prior admission, and hospital bed days (one bed day indicates admission and discharge on consecutive dates). Financial savings were evaluated as differential change in hospital claims between the study groups. All records without a valid admission and discharge date were excluded from analyses. Hospital visits that did not involve an overnight admission were excluded from utilization analyses.

Zero inflated negative binomial multivariate models were used to estimate treatment effect on the following dependent variables from individual claims record counts: hospital admissions; readmissions; and hospital bed days. Control variables in models included gender, age group, CEM weights, base admit count, and disease status for CAD, HF, diabetes, COPD, asthma, depression, chronic kidney disease, end stage renal disease, and cancer. Exponentiation of the intervention variable coefficient estimate was conducted, using the comparison group as the reference, to produce adjusted relative risk estimates. Multivariate logistic regression using the same control variables was conducted to estimate the relative likelihood of a treatment group member having an event (admission or readmission) relative to comparison members; results are presented as adjusted odds ratios and 95% confidence intervals. Gross financial savings for each intervention year were evaluated using general linear modeling of the difference in claims cost (all hospital claims paid by HCF) from the base period to each specific intervention year while controlling for base cost and additional covariates delineated above. A differenced cost variable (base to evaluation year) was selected as the dependent variable to conform to the linear statistical method being used and the accompanying assumptions. Savings values were determined by comparing change in costs of treatment group to the comparison group. All data manipulation and analysis, including CEM, was performed using SAS 9.2 software (SAS Institute Inc., Cary, NC).

## Results

Evaluation of balance between study groups and loss of initially eligible members after CEM matching is presented in Table 1. The CEM process resulted in pruning just 17 treatment members (0.3%) and 797 comparison members (2.7%) from eligible populations. An L1 post-match statistic of 3.57 E^−15^ compared to the pre-match statistic of 0.2072 is indicative of a high quality match with minimal imbalance between the resulting treatment and comparison study groups. Descriptive characteristics of final study groups displayed in Table 2, adjusted using CEM weights, confirm the comparability of treatment and comparison groups in regard to most demographic characteristics and medical conditions. Some differences remain in variables not used in matching, which were adjusted for in statistical testing for each subsequent analysis.

**Table 1:** **Study group sizes and balance metrics before and after matching using CEM**

	**Study group**	**Treatment**	**Comparison**	CEM L1 metric*
Before CEM match	34,282	4,965	29,317	0.2072
After CEM match	33,468	4,948	28,520	3.57 E^−15^
Members lost due to match	814	17	797	

*L1 metric is an indicator of balance/equivalence between study groups. A value of 1 indicates complete dissimilarity (no overlap) and a value of 0 indicates groups are perfectly equivalent or balanced.

**Table 2:** Demographic comparison of study groups, adjusted*

**Demographic variable**	**Treatment**	**Comparison**	**P value**
N	4,948	28,520	
% female	42.4%	42.4%	NS^‡^
average number of chronic core conditions (SD)	1.39 (0.64)	1.39 (0.64)	NS^†^
average base year total dollars	$4,590.32	$4,115.37	<0.01^†^
% with diabetes	43.9%	43.9%	NS^‡^
% with coronary artery disease	72.6%	72.6%	NS^‡^
% with heart failure	10.3%	10.3%	NS^‡^
% with COPD	2.9%	2.6%	NS^‡^
% with asthma	3.0%	2.1%	<.0001^‡^
% with chronic kidney disease	2.2%	2.1%	NS^‡^
% with end stage renal disease	0.3%	0.6%	<0.05^‡^
% with depression	0.8%	2.2%	<.0001^‡^
% with cancer	3.3%	2.9%	NS^‡^
Base admission rate (per 1000)	477.2	471.4	NS^†^
Base bed days (per 1000)	2,368.2	2,279.3	NS^†^
Base readmission rate (per 1000)	40.4	36.8	NS^†^
Age Groups	**Treatment**	**Comparison**	
20 - 29	0.1%	0.1%	NS^‡^
30 - 39	0.4%	0.4%	
40 - 49	2.1%	2.1%	
50 - 59	11.6%	11.6%	
60 - 69	28.3%	28.3%	
70 -79	35.4%	35.4%	
80 - 89	22.1%	22.1%	

*Comparison group statistics are adjusted for CEM weights.

SD – standard deviation, NS – not significant at .05, ‡ − Chi square test, † − T test.

Adjusted incidence rate ratios showed significantly lower rates of admissions, readmissions, and hospital bed days in the treatment group in contrast to the comparison group (Table 3). Base year comparisons for all three outcomes displayed no significant difference between study groups, further indicating match fidelity. Combined year models showed average relative reductions of 11.4% in admissions, 36.7% in readmissions, and 17.2% in bed days for the treatment group. Additionally, study group comparisons in each of the four intervention years displayed significant average reductions in the treatment group for each of these outcomes. A progressive trend of increasing reduction in utilization over time was noted for admissions and bed days.

**Table 3:** **Treatment effect on incidence of hospital utilization**

**Total admissions**					
**Period**	Relative risk*	**% Reduction**	**95% CI**		**P value**
All Years: 2010-2013	0.886	−11.4%	−14.3%	−8.4%	<.0001
Base	0.960	−4.0%	−9.0%	1.3%	0.1347
2010	0.937	−6.3%	−12.1%	−0.1%	0.0451
2011	0.891	−10.9%	−16.2%	−5.2%	0.0003
2012	0.875	−12.5%	−17.4%	−7.2%	<.0001
2013	0.869	−13.1%	−17.8%	−8.2%	<.0001
**Total readmissions**					
**Period**	Relative risk*	**% Reduction**	**95% CI**		**P value**
All Years: 2010-2013	0.633	−36.7%	−44.8%	−27.5%	<.0001
Base	0.940	−6.0%	−23.4%	15.4%	0.5538
2010	0.480	−52.0%	−64.0%	−35.9%	<.0001
2011	0.616	−38.4%	−52.8%	−19.6%	0.0004
2012	0.696	−30.4%	−46.5%	−9.6%	0.0066
2013	0.767	−23.3%	−40.4%	−1.2%	0.0397
**Total hospital bed days**					
**Period**	Relative risk*	**% Reduction**	**95% CI**		**P value**
All Years 2010-2013	0.828	−17.2%	−21.3%	−12.8%	<.0001
Base	0.969	−3.1%	−8.7%	2.8%	0.2969
2010	0.860	−14.0%	−21.8%	−5.3%	0.0022
2011	0.859	−14.1%	−21.8%	−5.7%	0.0015
2012	0.823	−17.7%	−24.7%	−10.1%	<.0001
2013	0.833	−16.7%	−23.3%	−9.6%	<.0001

*Adjusted incidence rate ratio (relative risk) estimated from zero inflated negative binomial models controlling for gender, age group, CEM weights, base admit count, and disease status for coronary artery disease, heart failure, diabetes, COPD, asthma, depression, chronic kidney disease, end stage renal disease, and cancer.

Logistic regression analysis using the entire intervention period indicated that the treatment group was significantly less likely to experience one or more admissions or readmissions than the comparison group, with adjusted odds ratios of 0.73 (95% CI 0.69-0.78) and 0.56 (95% CI 0.48-0.63), respectively (Table 4). Changing the reference group, the adjusted odds of having one or more admissions during the intervention period was 1.4 times higher in the comparison group relative to the treatment group (adjusted OR = 1.4, 95% CI 1.3-1.5). Similarly, the adjusted odds of experiencing one or more readmissions during the study intervention for the comparison group were 1.8 times higher than those of the treatment group (adjusted OR = 1.8, 95% CI 1.6-2.1).

**Table 4:** **Treatment effect on likelihood of hospital utilization**

**All years combined 2010 - 2013**	Odds ratio* treatment to comparison	**95% CI**	**Interpretation**
Admissions	0.73	0.69 - 0.78	27% decrease in odds of admission in Treatment group
Readmissions	0.55	0.48 - 0.63	45% decrease in odds of readmission in Treatment group

*Odds ratios from logistic regression models controlling for gender, age group, CEM weights, base admit count, and disease status for coronary artery disease, heart failure, diabetes, COPD, asthma, depression, chronic kidney disease, end stage renal disease, and cancer.

Financial savings in the treatment group as a result of participating in the MHG program are presented in Table 5. The average member savings are shown to increase over time, and reached nearly $1,500 in the 2013 intervention year. Cumulative average savings across the entire 4 year intervention were estimated to be $3,549.

**Table 5:** **Average member financial savings for each intervention year**

Years of cost evaluation*	**Treatment group cost change (AU$)**	**Comparison group cost change (AU$)**	**P value** ^**‡**^	Savings – PMPY** ^**†**^ **(AU$)**
Base to 2010	-$79.18	$389	0.0027	$468
Base to 2011	$241.70	$833	0.0009	$591
Base to 2012	$422.86	$1,440	<.0001	$1,017
Base to 2013	$819.12	$2,291	<.0001	$1,472
			Total	$3,549

PMPY, per member per year.

*General linear model controlling for gender, age group, CEM weights, base admit count, base cost, and disease status for coronary artery disease, heart failure, diabetes, COPD, asthma, depression, chronic kidney disease, end stage renal disease, and cancer.

** Gross savings prior to risk equalization pooling.

‡ − two-tailed T test.

† − per member per year.

## Discussion

The MHG program is a scalable long-term strategy to benefit the health of HCF members, while improving the sustainability of the health fund in the face of an aging and increasingly disease-burdened population. The current study validates the success of this strategy relative to these goals. Longitudinal program outcomes demonstrate that MHG members have realized significant overall reductions in hospital utilization during the 4-year evaluation period relative to matched non-participants. Significant reductions were also achieved in each of the 4 intervention years for all utilization outcomes, with the magnitude of program effect increasing over time with respect to avoided admissions and bed days. Together, study results support that participation in the MHG program led to the reduced frequency and length of hospital stays, the latter result suggesting a reduction in severity among hospitalizations that were not avoided entirely.

The trend of reduced utilization among MHG members is mirrored in the increased savings observed as participants are exposed to the program longer. Although claims costs are subject to a variety of influencing factors, the similar decreasing trends in utilization, which should be reflective of costs influenced by the program, lend support to MHG participation as the cause of savings. Savings represent only HCF claims; additional savings that may have accrued to publically-funded health care services such as emergency room visits were not available for assessment. As a consequence, the savings result from this study likely represents just part of the total possible savings since MHG can also impact utilization of public health services.

A primary strength of CEM as a matching method is the exclusion of relatively few treatment members in order to obtain unbiased and more generalizable study results. Health promotion programs can be more attractive to health plan members who are having current challenges with their own health where they may have experienced a recent adverse event. This phenomenon can lead to a treatment group made up of higher disease severity members, on average, than non-participants. Comparison statistics in this study suggest that the CEM matching and weighting process resulted in a treatment-to-comparison group match of adequate fidelity, resulting in study groups with no significant differences in any of the base hospital utilization rates and most of the disease conditions taken in-total as a representation of an overall level of disease morbidity. Any remaining group differences after CEM were accounted for by inclusion of available covariates in subsequent study modeling. Base hospital costs were one such variable, as a small but significant difference remained after adjustment for CEM weights, though the low magnitude difference was unlikely to drive differential regression-to-the-mean effects, which can bias group comparisons.

Prior evaluation of the MHG program demonstrated significant reductions in hospital admissions and 30 day readmissions at 12 and 18 months of program participation, with an increasing trend as time in program increased [13]. Present study results confirm prior conclusions and demonstrate that program benefit continues to increase over an extended period. Additionally, this result is supported by other studies that have reported on managed populations where intervention effect on hospital utilization has increased over time [18-22].

For a health and well-being program to positively impact the health of a covered population, it must be able to keep members engaged and receptive to the adoption of healthy behaviors that prevent avoidable medical utilization. The MHG program has exhibited a strong record of retaining enrollees. Of all MHG participants with CAD, HF, or diabetes who have enrolled over the course of the program, over 83% were still enrolled at the most recent evaluation end point (December 2013). This level of sustained enrollment indicates a relatively low dropout rate for any reason (either disenrollment from MHG, termination of HCF coverage, or death). Furthermore, the high rate of sustained and long-term participation in the program supports the generalizability of the study results in that continuous enrollment is typical of MHG members, not a rare or outlier characteristic.

MHG program outcomes, which demonstrate an early and increasing effect on hospital claims, have precedence in the literature. Some changes in health related behavior can have direct effects on health costs. Some intervention components can have a more immediate impact by avoiding acute hospital utilization. Improved adherence to important medications targeted at specific disease (i.e. heart failure medications) or improving the member’s ability to recognize physiological signs that often precede acute events and hospital admission are two such examples [23-25]. However, some changes in lifestyle and health behavior may not immediately manifest as a direct reduction in member health care consumption. For example, improving diet, long-term adherence to maintenance medications, and regular exercise will likely have a positive effect on participant’s physiology that will eventually be reflected in improved clinical indicators that represent long-term risk of hospitalization. A recent quasi-experimental study of a pharmacy coaching program illustrates this fact; participants had significantly increased medication adherence rates and significant reductions in blood pressure, lipid levels, and hemoglobin A1c test results, however minimal change in health-related costs compared to controls [26].

Increases in savings over time from disease management programs have been attributed by Nyman et al., who evaluated the sources of savings in a health promotion program, to the length of program enrollment relative to member capability to gain and apply new knowledge and techniques to avoid hospitalizations as well as the durability of the disease management effect, which can have a year-to-year compounding effect on savings as members continue to be covered by the insurance plan. They point out that disease management programs “should be viewed as investments that generate savings over the long-term” [27].

The results of this study should also be considered in the specific context of Australia, where health expenditure is rising sharply. In 2012–13, expenditures were estimated at $147 billion, compared to $69 billion just a decade before [28].

With the aging population, maintaining younger, healthier individuals in the insured population is necessary for insurance to remain affordable and for viability of community rated premiums [11]. However, the present system creates a disincentive to providing programs that are designed to improve the health and decrease hospital utilization of covered members. Specifically, savings from health improvement programs initiated by private health funds are ‘shared’ with other funds through a risk equalization pool. This proportion of shared savings is high for programs that enroll an older population due to the association of age with accumulated health risk and morbidity due to the age-based system of pooling claims costs. For example, over 75% of costs (and thus savings) are shared for members age 75 and older. Analyses of the state-based risk equalization pools for the MHG population over time have revealed that approximately 46% of insurance benefits for MHG participants are compensated by the age-based risk equalization system [HCF internal analysis]. Translated to savings resulting from MHG, HCF retains only 54% of the savings from the long-term program that it has implemented and maintained. Given the importance of reducing current health and cost trends for the sustainability of the current private health insurance system in Australia, the limited benefit to insurers who actively implement disease management programs can dissuade efforts to impact these trends. In effect, this system creates a disincentive for efforts toward health improvement, which benefits neither population health status nor the cycle of increasing health care costs of these populations.

### Limitations

Australian privacy laws and the dual public/private insurance system in Australia limited the availability of data that could be used for this study. Access to only those claims incurred in the private system precluded a complete picture of participants’ hospital utilization and cost. Study groups are well-matched with respect to available data; however, this assessment is subject to the limitation of omitted variable bias as a result of data limitations.

A primary limitation of non-experimental studies is the potential for selection bias. The retrospective design of the study and evaluation in the context of actual operating procedures precluded the use of a randomized design. However, rigorous quasi-experimental effectiveness studies in context can provide more realistic and generalizable results than a highly controlled design [29]. CEM matching of treatment and comparison members was accomplished to create more equivalent study groups and mitigate intergroup selection bias and variance to the extent possible using available data. Very few significant differences remained between the groups after matching, and the groups were statistically equivalent with respect to prior hospital utilization measures, chronic disease count, age, gender, and most conditions. A small, but significant difference in base cost remained; the treatment group was slightly higher cost. Together with the trend toward higher utilization (though non-significant) suggests that the treatment group may have been slightly higher in disease severity, which can minimize our ability to detect a significant effect.

Finally, regression to the mean can often account for differences between nonrandomized study groups if matching does not create equivalence in utilization and cost in the base period. As discussed above, the small remaining difference in cost between the groups at base was unlikely to result in differential regression. Furthermore, the increasing effect size over time indicates that regression to the mean [which is most predominant immediately after a point of high cost] was not a significant factor in accounting for the reduced hospital utilization and cost outcomes for the treatment group.

## Conclusions

Results demonstrate the longitudinal value of the MHG program. The 4-year evaluation shows the program is effective in the longitudinal mitigation and avoidance of adverse medical events leading to unnecessary or lengthy hospitalization for individuals with heart disease or diabetes. Program participation is associated with significant reductions in utilization and cost in the first year and the magnitude of these outcomes increase with time, corroborating utilization reductions demonstrated in the prior published program evaluation. Overall, the MHG program is an effective and scalable Australian CDMP to avoid unneeded hospitalization and associated costs. This study supports greater adoption of programs such as MHG as a means to control escalating health care costs. Expanded adoption, however, may be contingent upon policy changes, such as modification to the risk equalization system, that increase incentives for insurers who implement programs to improve population health and consequently bend the curve on health care cost escalation.

## Abbreviations

MHG: My Health Guardian
HCF: Hospitals Contribution Fund of Australia
CEM: Coarsened exact matching
BHC: Broader health cover
CDMP: Chronic disease management program
OR: Odds ratio
CI: Confidence interval
SD: Standard deviation
CAD: Coronary artery disease
HF: Heart failure
COPD: Chronic obstructive pulmonary disease
ESRD: End-stage renal disease
